# The Medical Schools

**Published:** 1903-09-05

**Authors:** 


					The Medical Schools.
The choice of a medical school is a matter that
should receive a great deal of attention at the hands
of the parents, guardians, or advisors of intending
medical students. Medical schools vary considerably
in their tone, in their traditions, in the quality of
instruction provided, and in many other ways, and
the ultimate success in life of an intending medical
practitioner may hang to a large extent on the choice
of his school.
"While London will probably always occupy the
leading place in the United Kingdom as a centre of
medical education, it does not follow that it is
always the best place for a prospective medical
student. Several of the large provincial towns
possess flourishing medical schools and can claim as
their children some of the leading men in the pro-
fession, and, indeed, they possess many advantages
which are not found in the metropolis. The size of
a hospital is not of great importance for teaching
purposes ; of more consequence is the size of the
medical school. Other things being equal, a large
school is usually better than a small one, because it
is better able to supply well-equipped laboratories
and first-rate teachers owing to the greater number
of fees received.
It may be said that it is impossible to generalise
Taauch further than this on the question of the choice
of a school. Individual requirements are probably
the surest guides.
Under the head "composition fee " we have men-
tioned the sum required by each hospital and medical
school for all the classes, lectures, and hospital
attendance which are necessary for the ordinary
diplomas, but at each of the hospitals special
arrangements can be made for separate courses.
All that is here attempted is to give a more or less
general impression of the various medical schools ;
for further information and particulars applica-
tion should be made to the individual institutions
by letter addressed to " The Dean of the Medical
School."
LONDON MEDICAL SCHOOLS.
All the teaching hospitals in London are now
schools in the faculty of medicine of the University
of London, and provide complete courses of study
for the preliminary scientific, intermediate, and final
M.B. examinations of the University, as well as for
the final examinations of the Universities of Oxford,
Cambridge, and Durham, and all the examinations
of the Conjoint Board of the Royal Colleges of
Physicians and Surgeons, and the Society of Apothe-
caries.
In the case of a student about to enter at a
402 THE HOSPITAL. Sept. 5, 1903.
London medical school, the question of residence
may offer some difficulty. There are manifold dis-
advantages attaching to lodgings where a youth,
perhaps fresh from school, can live free from effective
supervision. The ideal thing perhaps is, if expense
be not a matter of great importance, for him to board
with some member of the teaching staff, as there
usually are some of the younger members who are
willing to take in resident pupils on suitable recom-
mendations. If such a course be impossible, and
there are no friends or relatives with whom the
student can live, he may decide to take quarters in
the residential college, if there happen to be one
attached to the medical school of his choice, or fail-
ing this to take rooms. At most of the teaching
hospitals registers are kept of suitable lodgings, and
it is advisable that use should be made of these
before any particular rooms are decided upon.
St. Bartholomew's Hospital Medical School.
St. Bartholomew's is the oldest and the second
largest hospital in London, and is, perhaps, the
leading medical school. It was founded in 1123 by
Rahere as a hospital for the sick, and was under the
control of the Priory of St. Bartholomew. The
hospital and its revenues on the dissolution of the
priory in 1537 came into the possession of Henry VIII.
This monarch, at the petition of some of the leading
citizens of London, refounded the hospital by Royal
charter and gave back the greater portion of its
formier revenues.
The records of the medical school date back to
1662, though the actual date of its commencement is
not known.
The hospital contains 670 beds, in addition to 70
beds for convalescent patients at Swanley, in Kent,
and therefore there is no scarcity of clinical material.
Several entrance scholarships are awarded annually,
and the total value of scholarships and prizes presented
every year amounts to upwards of ?790. There is a
college for resident students within the precincts of
the hospital for admission to which a recommenda-
tion from one of the medical officers of the hospital
is required.
All full and university students must become
members of the amalgamated clubs on their entrance
to the medical school. The subscription is eight
guineas for full students and six guineas for uni-
versity students. On payment of the above fee the
student becomes a life member of all the clubs, in-
cluding the Abernethian Society, cricket, football,
and other clubs. The club ground?10 acres in area
?is at Winchmore Hill, on the Great Northern
Railway.
The composition fee to the medical school for
students commencing their medical studies is pay-
able as follows :?30 guineas at commencement of
anatomical and physiological studies, and 30 guineas
annually for the five years of the medical curriculum.
On qualification at the end of five years a student is
not liable for any further fees and receives a per-
petual ticket. Should he fail to qualify in this time
the fee for further instruction and attendance on the
practice of the hospital is 10 guineas for six months.
There are smaller fees payable by university
students and for those who desire to attend special
courses only without becoming full students of the
hospital.
Charing Cross Hospital Medical School.
Situated in the very centre of London, this hospital
has perhaps an advantage over all other similar
institutions in the metropolis as regards accessibility.
It is within two minutes' walk of the Charing Cross
terminus of the South Eastern Railway and the
Charing Cross Station of the Metropolitan Railway,
while omnibuses connect it with every part of
London.
Extensive additions are at present in course of
construction, including new surgical wards, out-
patient department, operating theatres, and isolation
wards, which will increase the 150 beds which the
hospital now possesses to 220.
In addition there is a convalescent home contain-
ing 50 beds at Limpsfield in Surrey.
Owing to the central position of the hospital, a
great many accidents and emergency cases are brought
there for treatment.
The medical school occupies a separate building in
Chandos Street, immediately opposite to the hospital.
It was built in 1881 and enlarged 1889 owing to the
increase in the number of students attached to the
hospital. ?
Several entrance scholarships are offered annually
for competition, including the Livingstone Scholar-
ship of 100 guineas, founded in memory of the late
Dr. Livingstone, a former student of the school, and
the Epsom Scholarship, giving a free education to the
successful candidate, who must be a foundation
scholar of the Royal Medical Benevolent College,
Epsom, who has passed the Preliminary Scientific
(M.B.) examination of the University of London.
Missionary studentships are also obtainable by
clergymen through the Society for the Promotion of
Christian Knowledge.
There is a Students' Club, whose recreation ground
is at Eltham.
The composition fee?including subscription to
the students' club?is ?115.
St. George's Hospital Medical School.
Considerable interest has been attracted to St.
George's Hospital lately owing to the proposal to
remove the hospital from its present site at Hyde
Park Corner and rebuild it elsewhere. The question
has arisen on account of the necessity of extending
the area of the site in order to bring the hospital
buildings into line with modern requirements, and,
as it is quite impossible to obtain the necessary land
in continuity with the present site, a removal to a
new and larger site is being contemplated by the
authorities.
The hospital contains 350 beds, of which 205 are
devoted to surgical and 146 to medical cases, in
addition to which there is a large convalescent home
of 100 beds at Wimbledon.
There are several entrance and other scholarships,
notably one of ?100 in Arts, open to candidates who
have not commenced their first winter attendance at>
a metropolitan medical school, the subjects and
scope of the examination being the same as for the
matriculation of the University of London; and
another of equal value in Science, the subjects being
those of the Preliminary Scientific (1st M.B.) exami-
nation of the University of London.
There are numerous appointments open to the
senior students, and amongst otherB may be specially
Sept. 5, 1903. THE HOSPITAL. 403
mentioned the following paid appointments, ?which
are open to students after they have held house
office :?
Two Medical Registrarships * ... salary ?200 per annum
Surgical Registrarship ... ... ? 200
Curatorship of the Museum  ? 200
Assistant Curatorship   ? 100
Resident Obstetric Assistantship ... ? 50
Two Demonstratorships of Anatomy ? 50
Senior Anassthetist  ? 50
Two Junior Antesthetists   ? 30
The compostion fee is ?150.
Guy's Hospital Medical School.
Founded by Thomas Guy and opened as a hospital
in 1724, Guy's Hospital rapidly rose to a place in the
front rank of metropolitan charities. The medical
school dates from 1768, at which time it was agreed
to open the practice of the hospital to the pupils
attached to the neighbouring St. Thomas's Hospital.
This association continued till 1825, during which
period the two hospitals enjoyed the title of the
United Borough Hospitals. After this date the two
medical schools become distinct.
Guy's Hospital contains 586 beds, and a note-
worthy feature is the reservation of certain wards,
containing 40 beds in all, for cases of special clinical
interest.
There is a college which accommodates about 60
students, who are under the supervision of a resident
warden. The rooms are furnished and the cost of
residence is moderate.
There is a remarkably well-conducted students'
club, which, perhaps, accounts in great part for the
good fellowship that is so observable among Guy's
men. By paying an annual subscription of two
guineas any student of the hospital obtains the
privileges of membership of all the clubs, including
the students' club.
Situated as it is on the south side of the river
near London Bridge, the hospital is in a particularly
suitable place, and it ministers to a large population
ot the hospital-patient class; there is, therefore,
plenty of work to be done, and the house appoint-
ments are especially valuable.
The composition fees are 16 guineas for the first
twelve months or less period, and eight guineas for
each subsequent six months.
King's College Hospital.
The medical is only one of the many faculties for
which educational arrangements are provided at
King's College; nevertheless as a medical school
King's College Hospital ranks very high, the instruc-
tion in the various branches of science, especially in
anatomy and physiology, being probably unsurpassed,
in London at any rate.
The hospital, which contains 220 beds, is situated
in Portugal Street, Lincoln's Inn, and is about five
minutes' walk from the college. In connection with
the hospital is a convalescent home, containing 40
beds, at Hemel Hempstead.
There has been a good deal of discussion lately
about the desirability of moving the hospital to
another site, and it is quite possible that the
authorities will see their way to carrying out this
scheme in the near future, in which case it is
Probable that the new hospital will be the best con-
structed of such institutions in the metropolis, as it
will be the most up-to-date. - -
Several entrance scholarships are offered for com-
petition every year, including two of the value of
?25 per annum, each tenable for four years, and one
of ?100. There are several others of smaller value.
Medical missionary studentships are also obtainable
through the Society for Promoting Christian Know-
ledge.
There is an athletic club whose ground is close to
Wormwood Scrubs Station.
There is a medical common room at the hospital,
the annual subscription for membership being 10s.
The composition fee is ?135.
The London Hospital Medical School.
Of all the large general hospitals this is perhaps
the one that is brought most prominently before the
notice of the general public. It is the largest insti-
tution of its kind, and being situated in the East
End of London, it manages to do a vast amount of
work in the course of the year.
This fact is perhaps the greatest disadvantage
which it possesses as a medical school, for where
there is so much clinical material it may not always
be possible to devote so much attention to that
thorough and careful clinical examination of every
patient which it is essential should become a matter
of habit to everyone who is to be a successful
practitioner in after life.
Several entrance scholarships are offered annually.
The number of appointments open to qualified
students is large, and some of them are of consider-
able value owing to the opportunities which they
provide for attaining experience.
There is a Clubs Union, embracing the athletic
and all the other clubs, to which all students are
expected by the College Board to belong. The
recreation ground is at Highams Park, and special
arrangements have been made with the Great
Eastern Railway for tickets at reduced rates for
members of the union.
The composition fee is 120 guineas if paid in one
sum, or 130 guineas if paid in three annual instal-
ments. A reduction of 15 guineas is allowed in the
case of sons of medical men.
St. Mary's Hospital Medical School.
Situated as it is near Paddington Station, St.
Mary's Hospital is in a most convenient position for
those students who have friends living in the West
End ; moreover the hospital is in a very accessible
position from all parts of London owing to the
proximity of the Great Western, Great Central,
Metropolitan, District, and Central London Rail-
ways.
The hospital at present contains 281 beds, and the
Clarence wing, which is now under construction and
nearing completion, will furnish an additional 80
beds, as well as three new operating theatres, a
medical clinical theatre, an obstetric department for
cases of difficult labour, and a complete electrical and
ce-ray department.
Entrance scholarships of a total value of ?610 are
offered annually, and include one in natural science
of ?145 open to any student who has not completed
a Winter Session of medical study at a medical
school, and one of ?145 open to students from
404 THE HOSPITAL. Sept. 5 1903.
Epsom College, being the sons of medical men, who
have not completed a Winter Session of study at a
medical school. This scholarship is not awarded by
examination.
Candidates gaining entrance scholarships are
required to enter at once as perpetual students of St.
Mary's Hospital, and pursue their full courses of study
there.
The composition fee is ?140 if paid in one sum on
entering the Medical School, and ?145 if paid in
four instalments. On payment of the full composi-
tion fee, and provided that he obtains a registrable
medical qualification within seven years from the
date of registration, the student becomes a perpetual
pupil; and is entitled to unlimited attendance on the
practice of the hospital.
The Middlesex Hospital Medical School.
The hospital, which is in Mortimer Street, W.,
contains 340 beds, including special wards for children
and the diseases of women. There is a cancer wing
which has 40 beds, and special research laboratories,
and the hospital therefore offers unequalled oppor-
tunities for the study of this disease, both clinically
and pathologically.
A complete electrical and a;-ray department has
recently been provided in a temporary building which
has been erected in the court-yard of the hospital.
The hospital has the advantage of possessing a
residential college which faces on the hospital garden.
It is capable of accommodating about 30 students.
There is an amalgamated students' club, which all
general and dental students are required to join on
their entrance to the medical school. The subscrip-
tion is two guineas for general and one guinea for
dental students.
Several entrance and other scholarships and prizes
are offered annually.
The composition fee, if paid in one sum on entrance,
is 135 guineas. It may be paid, if preferred, in three
instalments, viz. :?
On entrance ... ... ... ... 60 guineas.
At commencement of second year ... 50 guineas.
At commencement of third year ... 35 guineas.
St. Thomas's Hospital Medical School.
St. Thomas's Hospital occupies the most imposing,
and possibly the most suitable, position of all the
hospitals in London, situated as it is on the south
bank of the river opposite to the Houses of Parlia-
ment, while on the other side of it is one of the
poorest and most populous districts of London.
The hospital was founded in the twelfth century,
and was dedicated to St. Thomas a Becket. At that
time it was situated in Southwark. In 1538 the
hospital fell into the hands of Henry VIII. as it was
the property of the Church. It was refounded and
endowed in 1553, its dedication being transferred to
St. Thomas the Apostle.
In 1862 its old site was acquired by the South-
Eastern Railway Company, and the hospital was
transferred to its present position. The new
hospital was opened in 1871 by her late Majesty
Queen Victoria.
It consists of a series of separate blocks, six of
which are devoted to the use of patients, one is
applied to administrative purposes, and one consti-
tutes the Medical School.
The hospital contains 602 beds.
' There are a good many scholarships and prizes in
connection with the school.
The various students' clubs were amalgamated in
1888. The annual subscription for membership of
the amalgamated clubs is ?2 10s. for the first three
years and 2 guineas for the years following. After
five consecutive subscriptions the student becomes a
life-member, provided he has obtained a registrable
qualification.
The composition fee includes an entrance fee of
20 guineas and an annual fee of 30 guineas, which is
due in advance on the first day of the term in which
the student enters and on the corresponding day of
each successive year, until a registrable qualifica-
tion has been obtained. A student who qualifies
within six months of the date on which his last-
annual composition fee became due is allowed a
rebate of 15 guineas.
University College Hospital Medical School.
University College, like King's College, is com-
pletely equipped for providing educational facilities
in a wide range of subjects, of which medicine is
only one. The medical school is very flourishing7,
and takes a high place among the similar institu-
tions in London.
The hospital is, through the generosity of Sir J.
Blundell Maple, Bart., being rebuilt, and it h
thought that the whole of the new hospital will be
in working order within twelve months. There will
be accommodation for nearly 300 in-patients, with
extensive and well equipped out-patient and special
departments.
Scholarships and exhibitions of the value of ?^800'
are offered for competition every year, including the
Bucknill scholarship of ?120 and two entrance
exhibitions of the value of 60 guineas each, the
subjects for which are chemistry, physics, botany
and zoology, two exhibitions of 80 guineas each in
anatomy and physiology, the Atchison Scholarship of
?110 (awarded for general proficiency), and many
others.
The composition fee for the curriculum required
for the M.R.C.S. and L.R.C.P. is 150 guineas, while
for the degrees of the University of London it is
165 guineas, payable as follows : for the preliminary
scientific course, 25 guineas ; for the intermediate,
60 guineas; and for the final M.B. course, 80 guineas,
"Westminster Hospital Medical School.
The "Westminster Hospital stands opposite to
Westminster Abbey.
It was founded in 1715, and first opened at a small
house in Birdcage Walk, under the name of the
" Publick Infirmary for the Sick and Needy." At
the time of its commencement the only hospitals for
the relief of the sick poor in London were St. Bar-
tholomew's and St. Thomas's.
"Westminster Hospital is notable in that it was
the first institution of its kind in London which was
founded by the voluntary contributions of the public.
After occupying several different sites it was esta-
blished where it at present remains in 1834.
There is accommodation for upwards of 200 in-
patients.
There is a students' club union the subscription
for membership of which is included in the general
fees.
The composition fee is 110 guineas.
Sept. 5, 1903. THE HOSPITAL. 405
London (Royal Free Hospital) School of
Medicine for Women.
The Royal Free Hospital is in Gray's Inn Road,
and the London School of Medicine for Women is in
Hunter Street, Brunswick Square, W.C.
There are 170 beds in the hospital, all of which
are available for clinical teaching.
There are separate departments for the various
branches of medical work, and arrangements have
been made for students to attend the practice of a
number of special hospitals, including one of the
fever hospitals of the Metropolitan Asylums Board
and one of the hospitals under the Asylums Board of
the London County Council.
There are a good many scholarships and prizes
offered annually in connection with the school, among
which may be mentioned the Bostock Scholarship in
science, the annual value of which is ?60. The
scholarship is tenable for two years, and is extended
two more years on the receipt, by the Reid Trustees,
of a satisfactory report of the scholar from the
authorities of the school.
Besides the various scholarships and prizes assist-
ance is offered by several societies to ladies who
intend to work subsequently as medical missionaries.
The composition fee for the entire course after the
Preliminary Scientific Examination for the M.B. of
the London University and the Royal University of
Ireland is ?125, and that for the Preliminary
Scientific Course is 33 guineas.
The composition fee for the course of the M.B.
Durham, for the diploma of the conjoint colleges of
Scotland, or the Society of Apothecaries, includitg
elementary science, is ?135.
PROVINCIAL MEDICAL SCHOOLS.
As we have stated above there are some advantages
which provincial schools possess over those of the
metropolis, and among them may be mentioned the
greater facility with which a degree can be obtained
from those provincial schools which are connected
with local universities. Of course the kudos attached
to the London degrees is high, but it should be
remembered that the examinations are very severe,
and failure to pass on one or two occasions may so
prolong the course as to entail considerable pecuniary
hardships and disappointment.
The provincial medical schools at which a com-
plete medical education can be obtained are those of
Cambridge, Newcastle, Liverpool, Manchester, Leeds,
Birmingham, Bristol, and Sheffield. Certificates of
attendance at any of these schools are accepted by
the Royal Colleges, and also by the Uni versity of
London.
The University of Birmingham (Faculty of
Medicine).
To obtain a medical degree in the University of
Birmingham it is necessary as a rule to spend the
first four years of the five years' curriculum in the
University, but the Senate has power of recognising
attendance at another university as part of the
attendance qualifying for these degrees, and of
recognising examinations passed at such other
university as exempting from the examinations in
chemistry, physics, and comparative anatomy. In
the case of such students at least three years must
be spent in attendance upon classes at the University.
The fifth year may be spent at any other school or
schools of medicine recognised by lhe University.
In order to obtain the degrees cf M.B. and Ch.B.
five examinations have to be passed. The first is in>
chemistry and physics ; the second in anatomy,
comparative anatomy, and physiology ; the third in
pathology and bacteriology; the fourth in forensic-
medicine, toxicology, and public health; and th&
fifth, or final, in medicine, surgery, midwifery-,,
gynecology, therapeutics, mental diseases, and
ophthalmology.
The General Hospital and the Queen's Hospital,
containing between them upwards of 400 beds, are
amalgamated for the purpose of clinical instruction,
which is carried on under the direction of the Birming-
ham Clinical Board.
The composition fee for the whole course of
lectures and instruction at the University is ?85,
besides which there is a composition fee for attend-
ance on the medical and surgical practice and the-
clinical lectures at both hospitals of ?42, making a
total of ?127.
University College (Faculty of Medicine),
Bristol.
Students of the college are now admitted to the-
clinical practice of the Bristol Royal Infirmary and
the Bristol General Hospital conjointly. The infirm-
ary and the hospital comprise between them a total1
of 470 beds, and both are provided with extensive
out-patient departments and special departments.
The practice of the Bristol Royal Hospital for Sick
Children and Women, which contains 104 beds, and
that of the Bristol Eye Hospital, with 40 beds, arer
also available.
The total number of beds open for clinical
instruction is therefore 614.
The composition fee for the lectures is 65 guineas*
and that for hospital practice is 35 guineas.
Special fees are charged for clerkships and dresser-
ships, and the total fees payable, including these^,
amount to 127 guineas.
University op Cambridge.
In order to obtain the degree of Bachelor of
Medicine a student must become a matriculated
student of the University, reside in the University
the required portion of each of nine terms, pass on
obtain exemption from the previous examination,
pursue medical study for five years, and pass three
examinations.
Instruction in clinical medicine, surgery, and mid-
wifery is given at Addenbrooke's Hospital, so that it
is possible to obtain a complete medical education at
Cambridge ; but the usual practice is for men, after
spending three yeaVs at the University and passing
their second examination in anatomy and physiology,
to go to London and finish their clinical work there ?
and this is no doubt the best course to pursue.
University College, Cardiff.
Since it was founded, in 1883, University College,
Cardiff, has prepared students for the Preliminary
Scientific examination of the University of London,
and for the corresponding examinations of other
licensing bodies. In 1893, Chairs of Anatomy and
Physiology and a Lectureship in Materia Medica
406 THE HOSPITAL. Sept. 5, 1903.
and Pharmacy were established, making it possible
to spend three out of the five years of the medical
curriculum at this school.
Arrangements have been made with the managing
committee of the Cardiff Infirmary to give students
of the college the privilege of attending this hos-
pital, which contains 180 beds.
The composition fee for the three years' course of
study for the Preliminary Scientific and Intermediate
Examination for the London M.B. is ?57 10s.
The Yorkshire College, Leeds.
This is one of the colleges of the Victoria Univer-
sity. Clinical instruction is given in the general
infirmary, which contains 480 beds, and is provided
with very well equipped special departments.
Students of the college also have access for in-
struction to the Leeds Public Dispensary, the City
Fever Hospitals, the Hospital for Women and
Children, and the West Riding Asylum at Wake-
field.
The school of medicine, which is particularly well
?equipped in every way, is separated from the in-
firmary by a street only, an arrangement which
facilitates attendance at both institutions.
The composition fee for university students who
have already taken the course of instruction for the
first M.B. examination, and for non-university
students who have taken out the required instruction
in chemistry, chemical physics, and elementary biology,
i3 64 guineas, and that, for the hospital practice is
40 guineas.
University College (Medical Faculty),
Liverpool.
Students may now enter for the degrees of the
University of Liverpool, or may study here for the
degrees and qualifications of various other licensing
bodies. The conditions as to the degrees of the
University of Liverpool are for the present the same
as those of the Victoria University.
The laboratories are exceedingly well equipped,
and the facilities for instruction are most complete.
The clinical work may be done at the Royal
Infirmary, which contains 300 beds, the David
Lewis Northern Hospital, where there are 200 beds,
the Royal Southern Hospital containing 200 beds, or
the Stanley Hospital with 104 beds.
The lying-in, eye and ear, children's, and other
special hospitals are also open to students, and
courses of instruction in tropical diseases can be
obtained at the Liverpool School of Tropical
Medicine. Fellowships, scholarships, and prizes of
over ?700 are awarded annually.
Owens College, Manchester.
This is one of the colleges of the Victoria Univer-
sity. The medical department of O wens College is
exceedingly well provided with facilities for teaching
the sciences on which the arts, medicine, and surgery
are based?namely, anatomy, physiology, pathology,
and materia medica.
Clinical instruction is provided at the Manchester
Royal Infirmary, which contains 290 beds, and which
has large out patient and casualty departments.
Associated with the Infirmary is a Convalescent
Hospital at Cheadle containing 13G beds. Women
students are admitted to the practice of the Infir-
mary on the same terms as men.
Three halls are licensed by the college for the
residence of students?namely, Dalton Hall, Victoria
Park, and Hulme Hall, Plymouth Grove, for men,
and Ashbourne House, "Victoria Park, for women
students.
The composition fee for the full course of medical
study required for the ordinary qualifications is ?10,
in addition to which the fee for hospital practice is
?i2.
University of Durham (College of Medicine,
Newcastle-upon-Tyne).
For students who intend to graduate at the
Durham University it is necessary that one of the
five years of professional education should be spent
in attendance at the College of Medicine, Newcastle-
upon-Tyne. During the year so spent the student
must attend the medical and surgical practice and
clinical lectures at the Infirmary, which contains
280 beds. No residence at Durham is necessary.
The remaining four years of the curriculum may be
spent at Newcastle-upon-Tyne or at one or more of
the recognised medical schools.
The composition fee for the complete course of
lectures is 72 guineas, and for the medical and
surgical practice of the hospital 25 guineas.
The University of Oxford Medical School.
A course of study is provided at Oxford for
students until they have passed the Second Conjoint
Examination or the First Oxford M.B. At the
Radclifie Infirmary instruction is given in connection
with the University Medical School, and the entire
course may be completed at Oxford, but as a rule
here, as at Cambridge, it is the usual practice for
men, after spending three years at the University
and passing their examinations in anatomy and
physiology, to proceed to London and complete their
clinical work in the larger field there open to them.
University College (Department of Medicine),
Sheffield.
Clinical instruction is given at the Sheffield Royal
Infirmary, containing 240 beds ; the Sheffield Royal
Hospital, with 125 beds ; the Jessop Hospital for
"Women ; and several special hospitals. In connec-
tion with the College there is now a complete Dental
Department where students are able to go through
their full curriculum.
The composition fee for the lectures is ?63, and
for hospital practice ?i5.
SCOTTISH MEDICAL SCHOOLS.
University of Edinburgh.
To obtain the Edinburgh medical degree it is
necessary to pass the Preliminary Examination or
its recognised equivalent, and to pass four profes-
sional examinations. The first is in botany, zoology,
physics, and chemistry ; the second in anatomy,
physiology, materia medica, and therapeutics ; the
third in pathology, medical jurisprudence, and public
health ; and the final in surgery, clinical surgery,
medicine, clinical medicine, and midwifery.
The degree of M.B. is not conferred separately
from the Ch.B. In order to obtain the degree it is
necessary to spend at least two of the five years of
medical study in the University of Edinburgh.
Sept. 5, 1903. THE HOSPITAL. 407
Clinical instruction can be obtained in the Royal
Infirmary, which contains nearly 800 beds ; Royal
Hospital for Sick Children, with 120 beds; Maternity
Hospital; City Fever Hospital; and the Royal
Morningside Asylum.
School of Medicine of the Royal Colleges,
Edinburgh.
This School of Medicine is composed of lecturers
licensed by the Royal Colleges of Physicians and
Surgeons, and also recognised by the University, who
lecture in several buildings near the Royal Infirmary.
The teaching is similar to that of the Scottish
Universities, and the. students receive similar certifi-
cates at the close of each session.
The facilities for clinical instruction are similar to
those provided for the University.
Medical College for Women, Edinburgh.
Precisely the same facilities for medical study are
given as to male students in the School of Medicine,
Edinburgh. Regulations, fees and arrangements are
the same. The lecturers are recognised by the
University of Edinburgh as giving courses admitting
to the degrees of M.B., Ch.B. Clinical instruction is
given in the Royal Infirmary and other hospitals
mentioned above.
University of Glasgow.
The whole course of study for the M.B., Ch.B., can
be passed in the Medical School of the University.
The regulations are similar to those given above
for Edinburgh. Clinical instruction is given at the
"Western Infirmary, which contains 420 beds, and
also at the lunatic asylum and various special hos-
pitals.
Queen Margaret College, Glasgow
(the Women's Department of the University).
This is an integral part of the University of Glas-
gow. The courses, regulations and fees are the same
as. men. The college provides a separate building,
with clasps-rooms, library and other teaching appli-
ances.^ The women have all the rights and privileges
L!flvef^y students, and do their clinical work in
the Royal Infirmary, where special wards are reserved
for their use, and in other special hospitals.
St. Mungo's College, Glasgow.
The college buildings are situated within the
grounds of the Royal Infirmary, the wards of which
are available for clinical instruction, as also are
those of the Western Infirmary. All the classes in
this college qualify for the diplomas of the English,
Scottish and Irish Conjoint Boards.
Anderson's Medical College, Glasgow.
The college is situated in Dumbarton Road, close
to the Western Infirmary and within four minutes'
walk of the University.
University of Aberdeen.
The regulations are practically the same as those
given for Edinburgh. Clinical instruction is obtained
in the Royal Infirmary, which contains 200 beds, in
the Sick Children's Hospital, and several other insti-
tutions, including the Royal Lunatic Asylum and the
City Fever Hospital,
University op St. Andrews (University College,
Dundee, and United College, St. Andrews).
In order to graduate, at least two of the five
years of medical study must be spent either at the
United College, St. Andrews, or at University
College, Dundee. The remaining three years may
be passed at other medical schools, but the whole
course may be completed, if preferred, at University
College, Dundee.
IRISH MEDICAL SCHOOLS.
The School of Physic in Ireland, Dublin.
This school is formed by an amalgamation of the
medical school of Trinity College and of the Royal
College of Physicians.
Clinical instruction is given by the staff of Sir
Patrick Dun's Hospital, and courses of clinical study
can be carried out at many of the other hospitals in
Dublin.
The Catholic University Medical School.
Complete courses of lectures, as required by the
examining bodiels, are provided.
For all further information about the school
application should be made to the Registrar.
The Schools of Surgery of the Royal College
of Surgeons in Ireland.
The Schools of Surgery are attached by Charter
to the Royal College of Surgeons, Dublin.
Their work is carried on within the College build-
ings, and is subject to the control and supervision
of the Council.
There are special rooms set apart for the use of
lady students.
Full information is to be had on application to the
Registrar, Royal College of Surgeons, Dublin.
Queen's College, Belfast.
The courses of study meet the requirements of the
Royal University of Ireland and other examining
bodies.
Clinical instruction is given at the Royal Victoria
Hospital and other hospitals.
Full information can be had on application to the
Registrar, Queen's College, Belfast.
Queen's College, Cork.
Students can arrange the courses which they
attend to meet the requirements of the University
of Ireland, the Conjoint Boards of London, Edin-
burgh, or Dublin, and other examining bodies.
Clinical instruction is given at the North and
South Infirmaries, each of which contains 100 beds,
and at other hospitals.
Further information can be had on application to
the Registrar, Queen's College, Cork.
Queen's College, Galway.
Clinical teaching is carried on in the Galway Hos-
pital, the Galway Fever Hospital, and the Galway
Union Hospital. For further information applica-
tion should be made to Professor Pye, Honorary
Secretary of Medical Staff, Galway Hospital.
408 THE HOSPITAL. Sept. 5, 1903.
MEDICAL SCHOOLS AND MEDICAL
QUALIFICATIONS FOR WOMEN.
The following are the qualifying bodies which
admit women as candidates for their degrees or
diplomas, viz., all the Universities of England with
the exception of the Universities of Oxford and
Cambridge ; the Universities of Scotland ; the Royal
Universities of Ireland ; the Society of Apothecaries,
London ; and the Conjoint Board of the Royal Col-
leges of Physicians and Surgeons of Scotland and
Ireland.
The regulations of the General Medical Council in
regard to medical education and examinations apply
to women exactly as they do to men.
With regard to place of study, women medical
students can obtain a complete medical education by
becoming students of the London School of Medicine
for Women, which is connected with the Royal Free
Hospital; the Medical College for Women, Edin-
burgh ; or Queen Margaret College, Glasgow (which
is now an integral part of the University of Glas-
gow), all of which are open to women only ) or they
may study at the Schools of Surgery of the
Royal College of Surgeons in Ireland, where
special rooms are set apart for lady students ;
or at one of the Queen's Colleges at Belfast,
Cork, and Galway. They can also enter at
the Universities of Edinburgh, Aberdeen, and St.
Andrews ; at the University of Birmingham ; at
some of the colleges of the Victoria University ; and
at the University of Durham College of Medicine
and College of Science which are situated in New-
castle-upon-Tyne. The medical classes of the Uni-
versity of St. Andrews are open to women on an
equal footing with men, and it may be arranged
either to take the first two years at United College,
St. Andrews, and the rest at Dundee, or the whole
five years may be spent at University College,
Dundee. The Dundee Royal Infirmary offers facili-
ties for clinical instruction to women students.
For English women students who feel equal to the
examinations of the London University, the London
School of Medicine for Women will be found
thoroughly suitable, or they may spend a portion of
their time at Newcastle after matriculating at the
Durham University and then finish their course in
London. The Secretary of the School of Medicine
for Women, 30 Handel Street, London, W.C., will
give every information.

				

